# N-Acetylcysteine Increases the Frequency of Bone Marrow Pro-B/Pre-B Cells, but Does Not Reverse Cigarette Smoking-Induced Loss of This Subset

**DOI:** 10.1371/journal.pone.0024804

**Published:** 2011-09-16

**Authors:** Victoria L. Palmer, Michele D. Kassmeier, James Willcockson, Mohammed P. Akhter, Diane M. Cullen, Patrick C. Swanson

**Affiliations:** 1 Department of Medical Microbiology and Immunology, Creighton University School of Medicine, Omaha, Nebraska, United States of America; 2 Department of Biomedical Sciences, Creighton University School of Medicine, Omaha, Nebraska, United States of America; 3 Osteoporosis Research Center, Creighton University School of Medicine, Omaha, Nebraska, United States of America; National Institute on Aging, United States of America

## Abstract

**Background:**

We previously showed that mice exposed to cigarette smoke for three weeks exhibit loss of bone marrow B cells at the Pro-B-to-pre-B cell transition, but the reason for this is unclear. The antioxidant N-acetylcysteine (NAC), a glutathione precursor, has been used as a chemopreventive agent to reduce adverse effects of cigarette smoke exposure on lung function. Here we determined whether smoke exposure impairs B cell development by inducing cell cycle arrest or apoptosis, and whether NAC treatment prevents smoking-induced loss of developing B cells.

**Methodology/Principal Findings:**

Groups of normal mice were either exposed to filtered room air or cigarette smoke with or without concomitant NAC treatment for 5 days/week for three weeks. Bone marrow B cell developmental subsets were enumerated, and sorted pro-B (B220^+^CD43^+^) and pre-B (B220^+^CD43^−^) cell fractions were analyzed for cell cycle status and the percentage of apoptotic cells. We find that, compared to sham controls, smoke-exposed mice have ∼60% fewer pro-B/pre-B cells, regardless of NAC treatment. Interestingly, NAC-treated mice show a 21–38% increase in total bone marrow cellularity and lymphocyte frequency and about a 2-fold increase in the pro-B/pre-B cell subset, compared to sham-treated controls. No significant smoking- or NAC-dependent differences were detected in frequency of apoptotic cells or the percentage cells in the G1, S, or G2 phases of the cycle.

**Conclusions/Significance:**

The failure of NAC treatment to prevent smoking-induced loss of bone marrow pre-B cells suggests that oxidative stress is not directly responsible for this loss. The unexpected expansion of the pro-B/pre-B cell subset in response to NAC treatment suggests oxidative stress normally contributes to cell loss at this developmental stage, and also reveals a potential side effect of therapeutic administration of NAC to prevent smoking-induced loss of lung function.

## Introduction

Cigarette smoking is known to adversely impact innate and adaptive immunity through its pro-inflammatory and immunosuppressive effects on host defense systems [1]. Most studies investigating the effects of smoking on the immune system are understandably focused on those components localized to the respiratory and circulatory system, as they are most directly affected by the compounds and particulates present in cigarette smoke. However, we have recently shown that developing B cells in the bone marrow are also affected by cigarette smoking: mice exposed to a mixture of mainstream and sidestream smoke for three weeks showed a significant reduction in the percentage of bone marrow B220^+^CD43^−^ B cells (which includes pre-B, immature, and mature B cells), but not B220^+^CD43^+^ B cells (which includes pre/pro-B and pro-B/pre-B cells) [2]. Cessation of smoking for six weeks largely reversed this outcome [2]. How and why B cells at this developmental stage are negatively affected by cigarette smoke exposure is unclear.

The pathological effects of cigarette smoking are thought to be mediated in part by oxidative stress that is caused both from the oxidants present in cigarette smoke [3], and the reactive oxygen species produced by leukocytes activated in response to smoking [4]. The role of oxidative stress in smoking-induced pathologies has prompted efforts to investigate whether compounds with anti-oxidant properties can be used therapeutically to counteract the oxidative stress associated with cigarette smoke exposure [5]. N-acetylcysteine (NAC) is a molecule that possesses anti-oxidant properties through its ability to directly neutralize reactive oxygen species (ROS) via interactions with its free thiol, and its indirect activity as a precursor to glutathione, which plays a major role in protecting against endogenous and exogenous sources of oxidative stress [5]. NAC has been used in both animal models and humans to ameliorate the effects of smoking on lung function [5]. NAC has also been used to counteract the effects of ROS that are thought to contribute to immunological senescence. Specifically, NAC administration in a murine model of premature aging [6] and in elderly women [7] has been reported to enhance lymphocyte chemotaxis and proliferation, but suppress lymphocyte adherence *ex vivo*. The effects of NAC treatment on lymphocyte development were not reported in these experiments. We therefore wished to test whether NAC administered during a smoke exposure regimen in mice could counteract the negative effects of smoking on B cell development under these conditions. We also sought to determine whether the loss of bone marrow B220^+^CD43^−^ B cells observed in mice exposed to cigarette smoke could be attributed to a smoking-induced arrest of cell cycle or an increase in apoptosis in this cell population.

Consistent with previous results, we found that smoking had the largest adverse effect (2-fold reduction) on developing B cells that are immunophenotypically defined as B220^+^AA4.1^+^CD43^−^CD19^+^CD24^+^sIgM^−^sIgD^−^, which includes pro-B and pre-B cells. We found that NAC treatment unexpectedly expanded the baseline levels of this pro-B/pre-B cell subset by about 2-fold, but did not attenuate the smoking-induced loss of this population. No significant effects of smoking on apoptosis or the cell cycle status of B220^+^CD43^+^ or B220^+^CD43^−^B cells was detected. Taken together, these data argue against oxidative stress as being a direct cause of smoking-induced loss of developing B cells. However, the unanticipated observation that NAC promotes expansion of pro- and pre-B cells indicates that developing B cells are normally lost through pathways activated in response to oxidative stress, and also reveals a potential side effect of therapeutic NAC administration to ameliorate smoking-induced pathologies.

## Results

### Overview of experimental design

**Figure 1:**
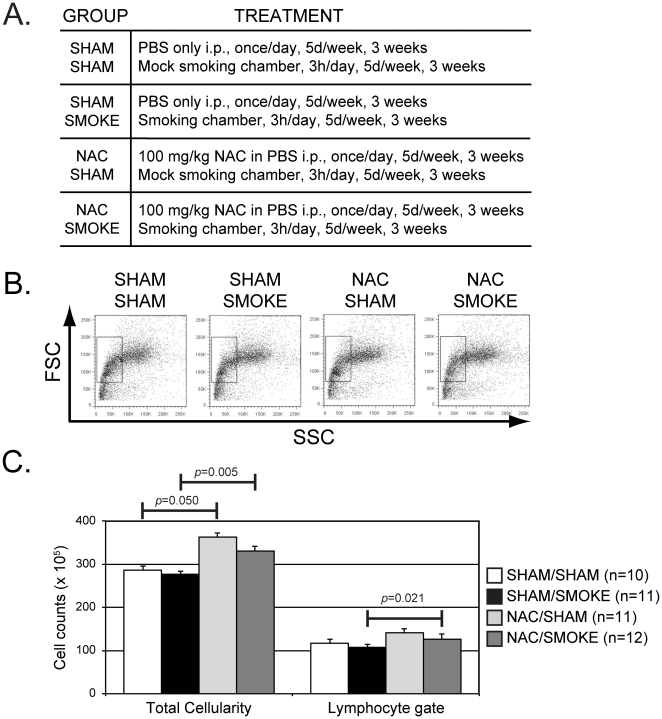
Total bone marrow and lymphocyte cellularities are increased by NAC treatment, but not smoke exposure. (A) Animals were divided into four groups and subjected to the treatments indicated. For additional details on experimental dosing, see Materials and Methods. (B) Bone marrow cells were analyzed by flow cytometry and a population with low forward scatter (FSC) and side scatter (SSC) properties enriched for viable lymphocytes was gated (Lymph+ gate). A representative plot is shown for an animal in each treatment group. (C) Mean values for the total bone marrow and gated lymphocyte cell counts were calculated for each of the experimental groups. Statistically significant differences (*p*<0.05) are indicated.

Wild-type adult C57BL/6 mice were divided into four treatment groups (n = 10–12 mice/group) in which animals received PBS or 100 mg/kg NAC in PBS and were then exposed to filtered air or a mixture of mainstream and sidestream cigarette smoke for 3 hrs. This regimen was repeated 5 days/week for three weeks as summarized in Figure 1A (sham/sham, NAC/sham, sham/smoke, and NAC/smoke, respectively). The duration of smoke exposure was chosen based on our previous study which demonstrated that mice exposed to cigarette smoke for three weeks showed a significant decrease in bone marrow B220^+^CD43^−^ B cells (which includes pre-B, immature B, and mature B cell subsets), but earlier precursor B220^+^CD43^+^ B cells (which includes pre/pro-B and pro-B subsets) and splenic transitional and mature B cells were not significantly affected by smoke exposure [2]. The route and dose of NAC administered to the animals was chosen based on studies showing that this treatment regimen was sufficient to reverse smoking-induced effects in other systems [8], [9]. When the experiment was completed, urine was collected to measure cotinine and creatine levels, and animals were euthanized and bone marrow harvested for B cell isolation and/or analysis using multicolor flow cytometry. The smoking procedure produced a mean value for the total suspended particles of 101±32 mg/m^3^ and a cotinine/creatine ratio of 1139±317 ng/mg 18 h after the last smoke exposure. Since the half-life of cotinine of 16 h [10], doubling the ratio to reflect the cotinine level immediately after smoke exposure yields a value that is >40-fold higher than the mean ratio observed in urine samples collected from human passive smokers, and is roughly equivalent to a moderate smoker who smokes 10–20 cigarettes/day [11].

Consistent with our previous results [2], total bone marrow cellularities and the total number of cells within a lymphocyte gate (defined based on the forward and side scatter gating strategy shown in Fig. 1B) were not significantly different between sham/sham and sham/smoke groups (Fig. 1C, Table 1). This was also true when NAC/sham and NAC/smoke groups were compared to each other. Unexpectedly, however, both NAC/sham and NAC/smoke groups showed a significant increase (27–38%) in total bone marrow cellularity compared to the sham/sham and sham/smoke groups. A similar, but smaller (21–36%) increase in the total number of cells within the lymphocyte gate was also observed, but the differences were only statistically significant when comparing the sham/smoke and NAC/smoke groups.

**Table 1:** Summary of total cell counts of various B cell subsets (×10^5^ unless indicated).

*Bone Marrow*	Sham/Sham	Sham/Smoke	NAC/Sham	NAC/Smoke	ONE WAY ANOVA^a^	Sham/Sham vs. Sham/Smoke^b^	Sham/Sham vs. NAC/Sham^b^	Sham/Smoke vs. NAC/Smoke^b^	NAC/Sham vs. NAC/Smoke^b^
	(n = 10)	(n = 11)	(n = 11)	(n = 12)					
Cellularity (×10^7^)	2.86±0.21	2.77±0.20	3.62±0.26	3.85±0.33	††	n.s.	n.s.	††	n.s.
Lymphs (×10^5^)	117±8	108±6	142±9	139±11	†	n.s.	n.s.	†	n.s.
Lymph^+^Ly6C^−^DX5^−^CD4^−^									
B220^+^AA4.1^+^	14.6±2.4	6.8±1.2	29.6±3.4	16.0±2.6	†††	†	†††	†	†††
Pre/Pro B and Pro-B (CD19^−^CD24^−^IgM^−^IgD^−^)	1.11±0.16	1.01±0.07	1.23±0.27	1.14±0.21	n.s.	n.s.	n.s.	n.s.	n.s.
Pro-B/Pre-B (CD19^+^CD24^+^IgM^−^IgD^−^)	9.96±1.85	4.12±0.91	21.0±2.4	11.5±2.0	†††	†	†††	††	†††
Lymph^+^Ly6C^−^DX5^−^CD4^−^									
B220^+^AA4.1^−^	10.9±1.2	9.59±0.71	9.35±0.71	8.32±1.03	n.s.	n.s.	n.s.	n.s.	n.s.
Pre- B (IgM^−^IgD^−^)	3.59±0.33	3.12±0.14	3.87±0.26	3.82±0.34	n.s.	n.s.	n.s.	n.s.	n.s.
Imm. B (IgM^+^IgD^−^)	0.54±0.07	0.47±0.04	1.05±0.09	0.73±0.09	†††	n.s.	†††	†	†††
Mature B (IgM^+^IgD^+^)	6.53±0.96	5.78±0.62	4.15±0.52	3.56±0.68	†	n.s.	†	†	n.s.

a Variance between groups by one-way ANOVA: n.s., not significant; †, *p*≤0.05; ††, *p*≤0.01; †††, *p*≤0.005.

b Post-hoc analysis by unpaired *t* test: n.s., not significant; †, *p*≤0.05; ††, *p*≤0.01; †††, *p*≤0.005.

**Figure 2:**
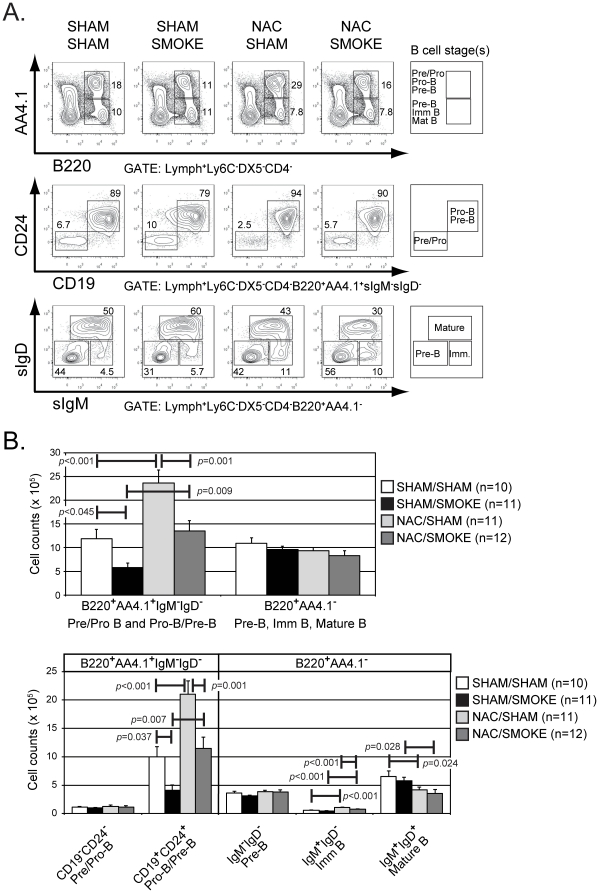
NAC treatment does not ameliorate the adverse effects of smoking on B cell development, but causes a smoking-independent 2-fold increase in the pro-B/pre-B cell subset. (A) Animals in the treatment groups indicated above the panel were analyzed for the expression of B220 and AA4.1 (top row), CD19 and CD24 (middle row), or sIgM and sIgD (bottom row) on bone marrow cells identified by the gating parameters shown below each row. Developmental subsets specified by the staining pattern are indicated at right with corresponding gates. The percentage of cells within the identified gate is shown for representative animals. (B) The absolute number of cells within each developmental subset identified in (A) was calculated for the animals in each treatment group (see Table 1). The mean values for each data set are plotted in bar graph format. Statistically significant differences between treatment groups (*p*<0.05) are indicated.

To enumerate B cells within various bone marrow developmental subsets, we originally intended to identify subsets based on differential B220 and CD43 staining as described previously [2]. However, the anti-CD43 antibody was inadvertently omitted from the antibody cocktail for one set of samples (representing 3–4 mice/treatment group). As a result, the experiment was underpowered for statistical analysis using CD43 as a marker. Despite this shortcoming, we nevertheless observed a substantial reduction in the mean number of developing bone marrow B220^+^CD43^−^ B cells in mice from the sham/smoke compared to the sham/sham group, whereas smoke exposure had smaller effect on the number of cells within the B220^+^CD43^+^ subset (Fig. S1). Similar results were obtained when NAC/sham and NAC/smoke groups were compared (Fig. S1). However, we also concomitantly stained for AA4.1, a marker that can be used as an alternative to CD43 to identify progenitor B cell populations with reportedly similar results [12]. Therefore, we used B220 and AA4.1 to segregate B cells into two populations, B220^+^AA4.1^+^ and B220^+^AA4.1^−^ (Fig. 2A, top row). Contaminating cells expressing DX5^+^, CD4^+^, and Ly6C were excluded from both populations, and sIgM^+^ and sIgD^+^ cells were additionally excluded from the B220^+^AA4.1^+^ subset [12]. Based on differential expression of CD19 and CD24, the B220^+^AA4.1^+^ population was further resolved into pre/pro-B (CD19−CD24^−^) and pro-B/pre-B (CD19^+^CD24^+^) subsets (Fig. 2A, middle row). The B220^+^AA4.1^−^ population, in turn, was subdivided based on differential sIgM and sIgD expression into pre-B (IgM^−^IgD^−^), immature (IgM^+^IgD^−^), and mature (IgM^+^IgD^+^) subsets (Fig. 2A, bottom row).

When sham/sham and sham/smoke treatment groups were compared, mice in the latter group were found to have about 50% fewer B cells in the B220^+^AA4.1^+^ fraction (*p* = 0.045), which was traced to a selective and significant reduction (∼60%) in the pre-B/pro-B (CD19^+^CD24^+^) subset (*p* = 0.037). The number of cells within each of the other developmental subsets analyzed was not significantly different between these treatment groups (Fig. 2B and Table 1). Interestingly, when sham/sham and NAC/sham groups were compared, we found that the latter mice showed a significant 2-fold increase in the number of cells within the B220^+^AA4.1^+^ population, which was again specifically traced to expansion of the pre-B/pro-B (CD19^+^CD24^+^) subset. By contrast, the absolute number of B220^+^AA4.1^−^ B cells was slightly diminished (but not significantly) in NAC/sham mice compared to sham/sham mice. However, within this subset, we observed a ∼2-fold increase in immature B cells (*p*<0.001), but this was offset by a 36% decrease in mature B cells (*p*<0.028). Interestingly, when NAC/sham and NAC/smoke groups were compared, we found that smoke exposure in NAC-treated animals had a quantitatively similar effect on pre-B/pro-B cells, as well as later subsets, as it did in animals receiving PBS only. Taken together, these results show that smoke exposure has a striking adverse effect on a pro-B/pre-B cell population, defined as B220^+^AA4.1^+^CD19^+^CD24^+^IgM^−^IgD^−^, and that concomitant NAC treatment does not negate the smoking-induced loss of this B cell subset, but does increase the frequency of these cells independent of smoke exposure.

**Figure 3:**
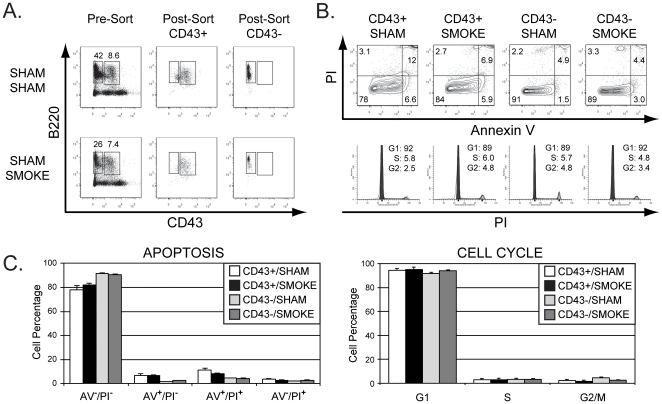
Apoptosis and cell cycle status in the B220^+^CD43^+^ and B220^+^CD43^−^ B cell subsets are not significant altered by smoke exposure. (A) B220^+^CD43^+^ and B220^+^CD43^−^ B cells were purified by FACS in the sham/sham and sham/smoke treatment groups (pre- and post-sort analysis, top row), and immediately stained with annexin V (AV) and propidium iodide (PI) (middle row) to identify early [AV^+^PI^−^] or late [AV^+^PI^+^] apoptotic cells or necrotic cells [AV^−^PI^+^], or stained with Vindelov's reagent (bottom row) to analyze cell cycle status. The percentage of cells within each quadrant or cell cycle phase is shown. Results are representative of 3–5 independent experiments. (B) Mean values for the data collected in panel A are plotted in bar graph format (C). No statistically significant differences between treatment groups were identified.

**Figure 4:**
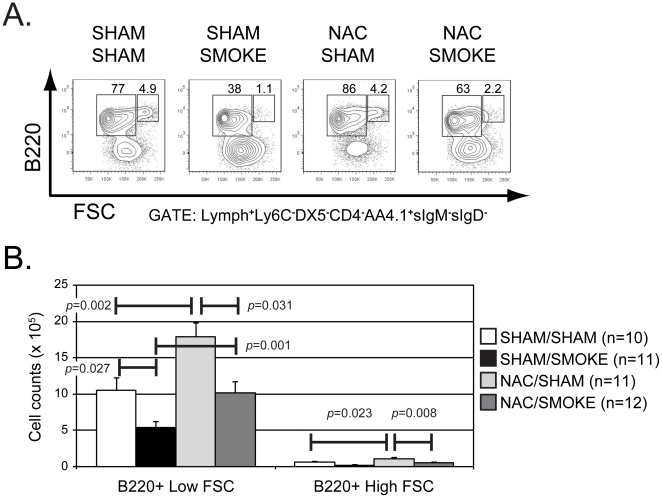
NAC treatment expands the number of both small and large B cells in the pro-B/pre-B cell subset. (A) The FSC profile of bone marrow B cells with a Lymph^+^Ly6C^−^DX5^−^CD4^−^B220^+^AA4.1^+^sIgM^−^sIgD^−^ immunophenotype were analyzed from animals in the indicated treatment groups. The percentage of B220^+^ cells within the low and high FSC gate is shown for representative animals. (B) Collected data is summarized as described in Figure 2B.

We speculated that the smoking-induced loss of pro-B/pre-B cells may be caused by an arrest of cell cycle progression or an induction of apoptosis following smoke exposure. To test these possibilities, we relied on our conventional strategy using B220 and CD43 staining. B220^+^CD43^+^ and B220^+^CD43^−^ B cells were sorted by FACS (Fig. 3A), and stained with either Annexin V and propidium iodide to evaluate the frequency of early and late apoptotic cells (defined as staining positive for Annexin V and either negative [early] or positive [late] for propidium iodide, respectively), or stained with Vindelov's reagent to examine the cell cycle status of sorted cells (Fig. 3B). We found that neither B220^+^CD43^+^ B cells nor B220^+^CD43^−^ B cells purified from mice in the sham/sham and sham/smoke groups showed any significant difference in the percentage of apoptotic cells or cells in the G1, S, or G2 phases of the cell cycle between treatment groups (Fig. 3C). Given this outcome, we considered the possibility that smoking might negatively impact proliferating B cells that represent a small fraction of this pro-B/pre-B cell subset more so than other cells within this population, and therefore the effects of smoking on cell cycle might be masked in this analysis. To investigate this possibility, we re-plotted the gated the B220^+^AA4.1^+^IgM^−^IgD^−^ population to visualize B220 staining versus forward scatter as an indicator of cell size (Fig. 4A). The B cells with high forward scatter (FSC) profile detected in this plot represent large pre-B cells expressing pre-BCR and are undergoing proliferative expansion [13]. We then reanalyzed the effects of NAC treatment and smoke exposure on B220^+^ cells within this population that were segregated based on a low or high FSC profile. We found that smoke exposure and NAC treatment had similar outcomes on both low and high FSC subsets that mirrored the effects observed in the B220^+^AA4.1^+^IgM^−^IgD^−^population overall (Fig. 4B).

## Discussion

In a previous study, we found that normal mice exposed to a mixture of mainstream and sidestream smoke for three weeks showed a significant reduction (∼50%) in the percentage of bone marrow B220^+^CD43^−^ B cells, but earlier B220^+^CD43^+^ B cell progenitors and splenic transitional and mature B cell subsets were not significantly affected by this smoking regimen [2]. Using a slightly different gating strategy, we have now further refined the developmental stage at which smoking affects B cell development, showing that three weeks of smoke exposure causes a significant and selective ∼60% reduction of B cells in a developmental subset that is phenotypically characterized as B220^+^AA4.1^+^CD19^+^CD24^+^IgM^−^IgD^−^ which includes pro-B and early pre-B cells. Other earlier and later developmental stages were not significantly affected by smoke exposure. These results contrast subtly to our earlier finding that the B220^+^CD43^+^ B cell subset, which also includes pro-B cells, was not significantly reduced by smoke exposure [2]. Since CD43 is known to be expressed on B cell developmental subsets that concomitantly express B220, AA4.1, CD19, and CD24 [14], we re-examined whether smoke exposure differentially affected B220^+^AA4.1^+^CD19^+^CD24^+^IgM^−^IgD^−^ cells that were either CD43^+^ or CD43^−^ within the group of animals in which cells were properly stained with both CD43 and AA4.1. Consistent with previous results [2], those in the sham/smoke and NAC/smoke group showed a selective reduction in the CD43^−^ fraction of these cells compared to their non-smoked counterparts. However, because of insufficient power (as discussed in the results), the differences were only statistically significant when comparing NAC/sham and NAC/smoke groups (Fig. S2).

We also tested whether the smoking-induced loss of developing B cells could be attributed to altered cell cycle progression or increased apoptosis. Although we did not find any significant differences in the frequency of cells in the various phases of the cell cycle or the percentage of cells undergoing apoptosis between sham- and smoke-exposed mice at the time when the animals were euthanized for analysis (which was 18 hours after the last smoking treatment), we cannot rule out the possibility that *in vivo* effects of smoke exposure on cell cycle and apoptosis in B cells are induced earlier than we had analyzed. Future experiments will address this possibility.

Because NAC has been investigated as a potential anti-oxidant therapy to attenuate the effects of smoking on lung function [5], we hypothesized that NAC treatment in mice could protect developing B cells from the adverse effects of smoking. However, we did not find this to be the case, but found unexpectedly that NAC-treated mice showed a significant (∼2-fold) increase the absolute number of B cells in the pro-B/pre-B cell (B220^+^AA4.1^+^CD19^+^CD24^+^IgM^−^IgD^−^) subset (Fig. 2B), especially the CD43^−^ fraction of these cells (Fig. S2), which is the same subset adversely affected by cigarette smoke exposure. This result suggests that the mechanism of smoking-induced loss of the pro-B/pre-B cell subset does not involve a direct response to oxidative stress in these cells, but may instead be a secondary consequence of the activation of pathways involved in this response in other cells. However, the observation that NAC treatment significantly increases the abundance of pro-B/pre-B cells in the absence of smoking suggests that oxidative stress normally contributes to the loss of cells within this developmental subset. During the window from pro-B to pre-B cell development, cells may encounter oxidative stress as they undergo apoptosis [5] or proliferation [13] in response to unproductive or productive heavy chain gene rearrangement. Although not specifically tested in developing B cells, NAC has been shown to inhibit apoptosis and potentiate proliferation in cell culture. Specifically, Rosati et al. reported that NAC inhibits the spontaneous apoptosis of human tonsillar B lymphocytes *in vitro* by inhibiting the processing of caspase-3 and -7 [15], and De la Fuente et al. showed that NAC enhanced the proliferation of leukocytes stimulated with concavalin A *in vitro* [16]. Since the number of B220^+^AA4.1^+^sIgM^−^sIgD^−^ B cells with low and high FSC profiles are both increased by NAC treatment, the evidence suggests NAC treatment does not uniquely affect an actively proliferating B cell subset (Fig. 4B). Determining how NAC promotes expansion of pro-B/pre-B cells in mice will be a focus of future investigations.

Because NAC is being investigated for therapeutic use, one implication of this study is that patients treated with NAC treatment may show evidence of bone marrow lymphocytosis. The physiological consequences of this potential side effect are unclear at this point, because the number of pro-B/pre-B cells that progress to subsequent stages of development may be limited by the physical availability of niches to support their continued maturation. However, if NAC is shown to promote survival of developing B cells by inhibiting apoptosis, this situation may enable B cells with unrepaired V(D)J recombination-induced DNA breaks to survive long enough to permit illegitimate and possibly oncogenic rearrangements.

## Materials and Methods

### Animals and experimental conditions

The animal research protocol described here was approved by the Creighton University Institutional Animal Care and Use Committee (investigator: D.M.C.; protocol number 882). Adult (4–5 month old) normal female C57BL/6 mice were weighed and randomized into four treatment groups (n = 10–12 mice). Animals were injected intraperitoneally with either PBS or 100 mg/kg NAC (10% solution, Roxane Laboratories, Columbus OH). Two hours later, animals were exposed to either room air or a mixture of mainstream and sidestream cigarette smoke derived from burning Kentucky 3R4F reference cigarettes (Tobacco and Health Research Institute, University of Kentucky) using a TE-10 smoking device (Teague Enterprises, Davis, CA) for 3 hours as previously described [2]. These treatments were performed 5 days/week for three weeks. The average total suspended particle (TSP) level created by this smoking regimen was 101±32 mg/m^3^. Urine was collected 18 h after the final treatment and cotinine and creatine levels were measured by ELISA as previously described [2]. Animals were then euthanized and bone marrow was collected and prepared for cell analysis or sorting using flow cytometry.

### Flow cytometry

Bone marrow cell suspensions were prepared as previously described [2], and stained with a cocktail containing the following mouse-specific antibodies obtained from BD Biosciences (San Jose, CA), eBioscience (San Diego, CA), or Southern Biotech (Birmingham, AL): FITC-anti-IgD (11-26c.2a); PE-Texas Red-anti-B220 (RA3-6B2); PE-CD93 (AA4.1); APC-anti-IgM (II/41); Alexafluor700-anti-CD4 (GK1.5), SpectralRed anti-CD24 (30-F1); PerCP-Cy5.5 anti-Ly6C (AL-21); PE-Cy7 anti-pan NK (DX5); APC-Cy7 anti-CD19 (1D3), and biotin anti-CD43 (S7). Biotinylated antibodies were revealed using a streptavidin conjugate to QDot 585 (Invitrogen, Carlsbad, CA). FACS was used to isolate B220^+^CD43^+^ and B220^+^CD43^−^ B cells from bone marrow preparations pooled from two animals within each of the sham/sham and sham/smoke treatment groups. Sorted cells were immediately analyzed for apoptosis by staining with Annexin V and propidium iodide using a commercially available kit according to the manufacturer's instructions (BD Biosciences), and evaluated for cell cycle status by with propidium iodide according to the method of Vindelov [17]. All flow cytometric data was analyzed using FlowJo (TreeStar, Ashland, OR) or ModFit LT (Verity Software, Topsham, ME).

## Supporting Information

Figure S1 **Effects of smoking and/or NAC treatment on B cells subsets identified by staining with B220 and CD43.** This shows the B220 vs CD43 staining profiles for bone marrow lymphocytes, and the calculated frequencies of B220^+^CD43^+^sIgM^−^sIgD^−^ and B220^+^CD43^−^ B cell subsets for animals in the four treatment groups.
(DOCX)

Figure S2 **Smoke exposure and NAC treatment have differential effects on the CD43^+^ and CD43^−^ fractions of the B220^+^AA4.1^+^sIgM^−^sIgD^−^CD19^+^CD24^+^ pro-B/pre-B cell subset.** This shows the calculated frequencies of the CD43^+^ and CD43^−^ fractions of the B220^+^AA4.1^+^sIgM^−^sIgD^−^CD19^+^CD24^+^ pro-B/pre-B cell subset for animals in the four treatment groups.
(DOCX)
