# Supplementary material for: N-Acetylcysteine Increases the Frequency of Bone Marrow Pro-B/Pre-B Cells, but Does Not Reverse Cigarette Smoking-Induced Loss of This Subset
Source: PLoS One. 2011 Sep 16;6(9):e24804. doi: 10.1371/journal.pone.0024804 (PMC3174966; doi:10.1371/journal.pone.0024804)
Supplement: Figure S1 — Effects of smoking and/or NAC treatment on B cells subsets identified by staining with B220 and CD43. This shows the B220 vs CD43 staining profiles for bone marrow lymphocytes, and the calculated frequencies of B220+CD43+sIgM−sIgD− and B220+CD43− B cell subsets for animals in the four treatment groups. (DOCX) [file pone.0024804.s001.docx]

**
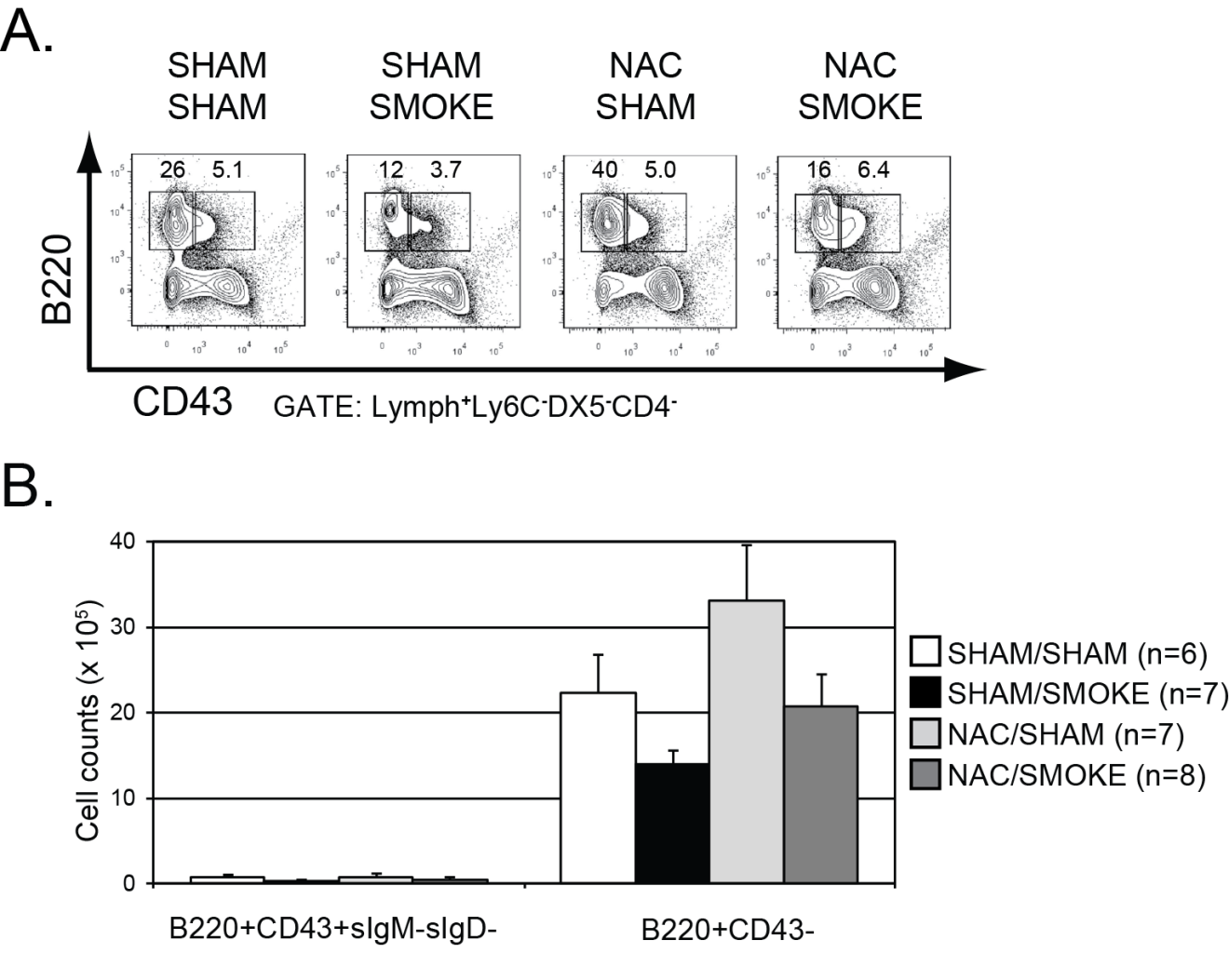
**

**Figure S1.** Effects of smoking and/or NAC treatment on B cells subsets identified by staining with B220 and CD43. (A) Bone marrow cells isolated from animals in the indicated treatment groups was analyzed for the expression of B220 and CD43 among Lymph^+^Ly6C^-^DX5^-^CD4^-^ cells. The percentage of cells within each of the identified gates is shown for representative animals. (B) The absolute number of cells within each developmental subset identified by the indicated immunophenotype (among Lymph^+^Ly6C^-^DX5^-^CD4^-^ cells) was calculated for the animals in each treat treatment group (n=6-8, as indicated at right). The mean values for each data set are plotted in bar graph format. No statistically significant differences between the treatment groups were obtained.
