# Supplementary material for: N-Acetylcysteine Increases the Frequency of Bone Marrow Pro-B/Pre-B Cells, but Does Not Reverse Cigarette Smoking-Induced Loss of This Subset
Source: PLoS One. 2011 Sep 16;6(9):e24804. doi: 10.1371/journal.pone.0024804 (PMC3174966; doi:10.1371/journal.pone.0024804)
Supplement: Figure S2 — Smoke exposure and NAC treatment have differential effects on the CD43+ and CD43− fractions of the B220+AA4.1+sIgM−sIgD−CD19+CD24+ pro-B/pre-B cell subset. This shows the calculated frequencies of the CD43+ and CD43− fractions of the B220+AA4.1+sIgM−sIgD−CD19+CD24+ pro-B/pre-B cell subset for animals in the four treatment groups. (DOCX) [file pone.0024804.s002.docx]

**
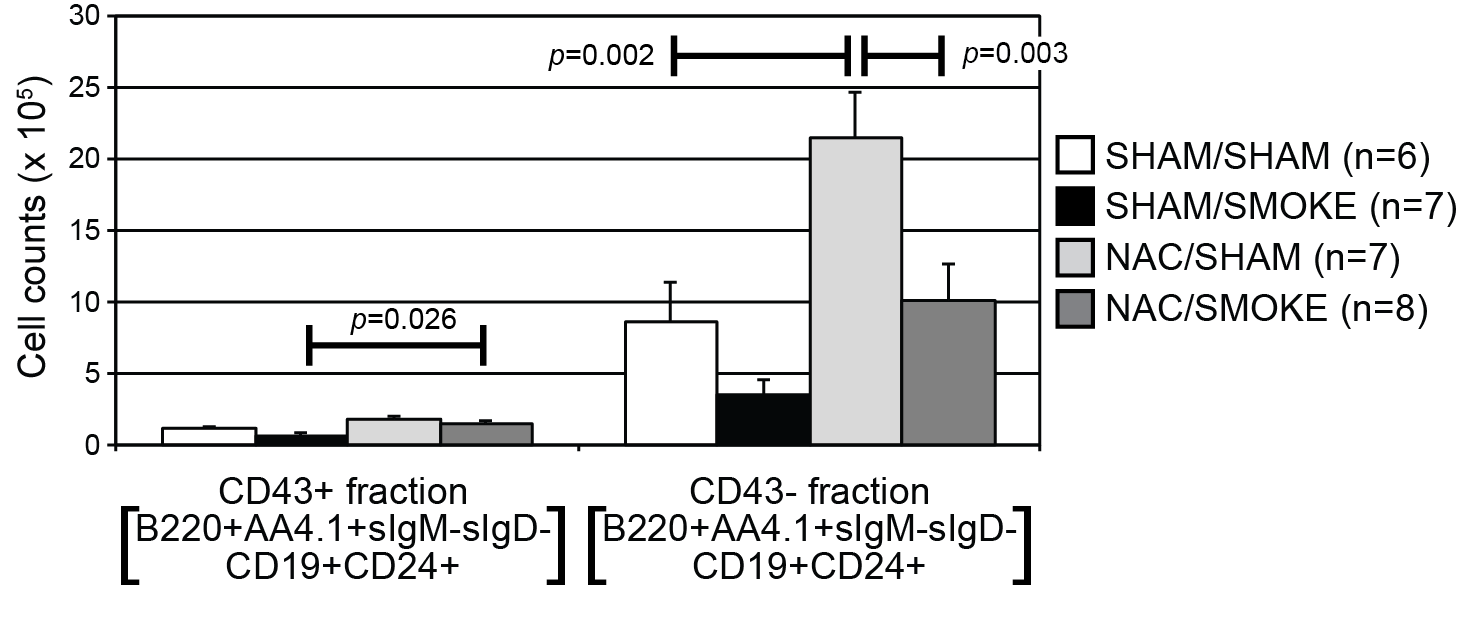
**

**Figure S2.** Smoke exposure and NAC treatment have differential effects on the CD43^+^ and CD43^-^ fractions of the B220^+^AA4.1^+^sIgM^-^sIgD^-^CD19^+^CD24^+^ pro-B/pre-B cell subset. B220^+^AA4.1^+^sIgM^-^sIgD^-^CD19^+^CD24^+^ pro-B/pre-B cells identified by the gating strategy shown in Figure 2A were analyzed for CD43 expression The absolute number of CD43^+^ and CD43^-^ cells within this population was calculated for the animals in each treatment group (n=6-8, as indicated at right). The mean values for each data set are plotted in bar graph format. Statistically significant differences between the treatment groups are indicated.
